# Levels of dietary diversity and its associated factors among children aged 6–23 months in West Shoa, Ethiopia: a comparative cross-sectional study

**DOI:** 10.1017/jns.2022.17

**Published:** 2022-03-17

**Authors:** Kefyalew T. Belete, Derese B. Daba, Seifadin A. Shallo, Mecha A. Yebassa, Kababa T. Danusa, Diriba A. Gadisa

**Affiliations:** 1 Department of Public Health, College of Medicine and Health Science, Ambo University, Ambo, Ethiopia; 2 Department of Midwifery, College of Medicine and Health Science, Ambo University, Ambo, Ethiopia; 3 Department of Pharmacy, College of Medicine and Health Science, Ambo University, Ambo, Ethiopia

**Keywords:** Comparative cross-sectional, Dietary diversity, Infant and young child feeding practices, Minimum meal frequency, Oromia, West Shoa zone, DDS, dietary diversity score, IYCF, infant and young child feeding, MAD, minimum acceptable diet, MMF, minimum meal frequency

## Abstract

Dietary diversity is one of the eight core indicators of infant and young child feeding (IYCF) practices. It is also a proxy for nutrient adequacy of the diet of individuals. There are minimal studies showing the level of dietary practice in urban and rural settings comparably. Hence, the present study intended to assess and compare differences in the level of dietary diversity and its contributing factors in urban and rural settings of the West Shoa zone of Oromia, Ethiopia. A community-based comparative cross-sectional study was conducted among 674 pairs of mothers/caregivers and children aged 6–23 months using a multistage sampling technique. Data were analysed and descriptive summaries were presented with tables, charts and graphs. A linear regression analysis was used to identify factors that were associated with the level of dietary diversity. The dietary diversity score (DDS) was 26⋅1 % (95 % CI 22⋅8, 29⋅5) both in urban and rural (*P* < 0⋅001), and also the minimum meal frequency was 56⋅5 % (95 % CI 52⋅7, 60⋅2) (*P* < 0⋅038). Child from merchant mother, own production of foods at the household level and frequent advice of IYCF practices during Post natal care (PNC) visit in urban residents, maternal secondary educational level, living with caregiver only, having a merchant father, advice of IYCF practice during PNC visit and utilisation of horse as a means of transportation in rural were positively associated with the level of dietary diversity. Generally, infant and young children who received the recommended dietary diversity and the minimum meal frequency were low in the study area both in the urban and rural settings.

## Introduction

Dietary diversity, one of the eight core indicators of infant and young child feeding (IYCF) practices, is a qualitative measure of food consumption that reflects household access to a variety of foods, and it is also a proxy for nutrient adequacy of the diet of individuals^(1)^. Adequate and diversified nutrition is essential in the first thousand days (conception to 24 months) for healthy growth, proper organ formation and function, a strong immune system, neurological and cognitive development^(2)^. On the contrary, inadequate consumption of high quantities and quality nutrients leads to child malnutrition. Child malnutrition in its different forms impacts the overall growth and development of a child^(1,3,4)^.

Malnutrition is currently thought to be the leading cause of the global burden of disease. Globally in 2019, an estimated 144 million children under 5 years of age or 21⋅3 % were stunted, whereas 47 million or 6⋅9 % were wasted of which 14⋅3 % were severely wasted. Moreover, children in Asia and Africa bear the greatest share of all forms of malnutrition^(3)^. Among the enormous contributing factor of malnutrition, inadequate quantity and quality diet during the first 2 years of life contribute to the lion's share.

World Health Organization's (WHO) IYCF practices advise exclusive breastfeeding (EBF) for the first 6 months of life followed by an introduction of solid, semisolid and soft foods at age of 6 months, then gradual increases in the amount of food given and frequency of feeding as the child grows older. It is also important for young children to receive a diverse diet, which includes foods from different food groups that satisfy children's growing micronutrient needs^(1,2,5)^.

Despite this recommendation, globally only 44 % of infants aged 0–6 months old exclusively breastfed between 2015 and 2020, and only less than a quarter of children aged 6–23 months meet the criteria of age-appropriate dietary diversity and feeding frequency^(6)^. According to a global nutrition report in 2017, globally only 15⋅6 % of children aged 6–24 months old ate a minimum acceptable diet (MAD), 68⋅5 % of infants aged 6–8 months old ate solid food in all forms and around 51⋅2 % of children aged 6–24 months old did not get the recommended minimum number of meals^(7)^. This report also indicated a significant difference in IYCF practice in an urban and rural setting. Moreover, It was indicated that rural residents are better in the initiation of EBF and continued breastfeeding at years 1 and 2, whereas urban residents are better at maintaining MAD, minimum meal frequency (MMF) and minimum dietary diversity (MDD)^(7)^.

Reports in Africa are showing that IYCF practice indicators are still measuring low and a significant variation in urban and rural settings was witnessed. A study from the demographic and health survey of Tanzania (TDHS-2015) indicated that only 26 % of children received recommended MDD^(8)^, whereas a demographic and health survey of Ethiopia (EDHS-2016) showed that only 8⋅5 % of children aged 6–59 months old had received a recommended MDD^(9)^. A systematic review of studies from 2011 to 2018 among children aged 6–23 months old in Ethiopia showed that the pooled prevalence of dietary diversity feeding practice among children aged 6–23 months old was 23⋅35 %^(10)^.

Studies in Ethiopia and the rest of the world showed several factors that would have increased IYCF practice indicators in general and dietary diversity in specific among children aged 6–23 months old. Increased maternal education, household monthly income, being urban resident, mothers’ knowledge on dietary diversification and attending any growth monitoring programmes as well as cooking demonstration, proper and sufficient exposure to media, antenatal and postnatal care service utilisation, a household visit by health extension worker (HEW), husband involvement in IYCF programmes and mothers’ decision-making ability and age of mother and child are some of the positively associated factors acquiring better and sufficient diet^(9–22)^. However, almost all of the studies attempted to find predictors (associated factors) of dietary diversity using maximum likelihood estimation that shows changes in the log odds of the dependent, not direct changes in the dependent itself. The studies also failed to show to what extent each variable contributes either in increment or reduction of levels of dietary diversity if intervention plans are implemented in the study setting. Furthermore, a majority of the studies were conducted in urban settings leading to minimal information regarding the level of dietary diversity in rural parts of the country (Ethiopia).

Several efforts have been made to alleviate problems associated with IYCF practices and their long-term complications nationally and internationally. Different policies and strategies were formulated that could enhance practices in EBF, dietary diversity and reduce malnutrition^(23–25)^. Despite these national and international policies and strategies, problems related to children's nutritional adequacies in terms of quality and quantities are minimal. These can be due to the mere effect of an insignificant number of studies showing discrepancies in an urban and rural setting. The present study intended to assess and compare differences in the level of dietary diversity and its contributing factors in an urban and rural setting of the West Shewa zone of Oromia, Ethiopia. Thus, the generated information will serve as input for designing an intervention plan to reduce the problem in the study setting. Furthermore, the generated information will be an input for local authorities, decision-makers, aid organisations and policymakers.

## Methods

### Study design and period

A community-based comparative cross-sectional study was conducted among 686 (343 from urban and 343 from rural) children aged 6–23 months in the West Shoa zone of Oromia region, Ethiopia from July to October 2019. The West Shoa zone has 2 058 676 population sizes, out of which 2⋅32 % (47 762) are children aged 6–23 months.

#### Source population

All children aged 6–23 months along with their mothers/caregivers who resided in the West Shewa zone.

#### Study population

The study populations were randomly selected children aged 6–23 months along with their mothers/caregivers who resided in randomly selected Woreda of the zone.

### Inclusion and exclusion criteria

#### Inclusion criteria

All children aged 6–23 months with their mother/caregiver in randomly selected woredas of the study area at least for the past 6 months were included.

#### Exclusive criteria

Those children who were sick before 1 week of commencement of the study were excluded. Critically ill mothers/caregivers who could not respond to the interviewer and mothers/caregivers with hearing impairment were also excluded from the study since they are a major source of information.

### Sample size determination and sampling procedures

The double population proportion formula was used to determine the sample size considering the following assumptions: 95 % confidence level, 5 % margin of error, 80 % power, *p*_1_ = 59⋅9 %, *p*_2_ = 17 % (*p*_1_ and *p*_2_ indicate the proportion of children with adequate dietary diversity in urban and rural, respectively)^(20,22)^, a design effect of 2 and non-response of 10 %.^(26)^
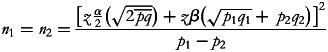
where 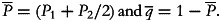


Then *n*_1_ = *n*_2_ = 156, multiplying by DE of 2 = 312, adding a 10 % non-response rate, the final sample size becomes 343 in each group. Thus, a total of 686 children (343 from urban and 343 from rural) were studied.

A multistage sampling technique was employed to select a representative sample from the study population. The first four woredas in the West Shewa zone, namely Gindeberet, Jaldu, Bako and Ambo, were selected randomly by a lottery method. Then in each selected woredas, two kebeles (one urban and one rural) in the Woreda/District were included in the study using a lottery method. For each kebele, the sample size was allocated proportionally to the total number of children aged 6–23 months who resided in the kebele. Finally, the study participants were identified by using a systematic random sampling method from each selected kebele using the family folder of HEWs as a frame. The first respondent from each kebele was chosen using the lottery method and the subsequent respondents were determined by the sampling interval (every *K*th).

### Variables

#### Dependent

The level of dietary diversity was measured as 0 to 7.

#### Independent

Socio-demographic variables were age, sex, educational status of family/caregiver, occupation of family/caregiver, family size and income.

#### Maternal and child health

Variables were sex and age of the child, birth order of the child, parity gravidity, antenatal and postnatal care follow-up, advice on IYCF practices and child's living arrangement.

IYCF practice variables were initiation of EBF, complementary feeding practice and MMF.

### Data collection methods, procedures and measurements

A structured, pre-tested, interviewer-administered questionnaire was used to collect the required quantitative information through face-to-face interviews with the child's mothers/caregivers. The questionnaire was composed of socio-demographic characteristics of children, obstetrics and health service characteristics of mothers and dietary diversity feeding practices. Moreover, data on dietary diversity were collected using the WHO indicators for assessing IYCF practices^(1)^. The dietary diversity data were collected using a 24-h recall method; that is, mothers were asked to recall all food items given to their child in the past 24 h before the survey. The consumed food items were then grouped under the seven food groups, namely grains, tubers and roots; legumes and nuts; fruits and vegetables; vitamin A-rich foods; eggs; flesh foods and dairy products. In order to determine the proportion of children with high or low DDS, a dichotomous variable was created based on food groups eaten per 24 h. Those children who had eaten food items from four or more food groups have been considered as having achieved DDS (high DDS). Those children who had eaten food items from three or fewer food groups have been considered as not achieving their DDS (low DDS). *MMF* was also measured based on frequencies of weaning per 24 h. A child was considered as received MMF when fed four or more times per day^(1)^.

In order to establish an association between dependent and independent variables, a discreet variable, namely level of dietary diversity with scores of 1–7, was used as a dependent variable.

### Data quality control

First, the questionnaire was prepared in English and then translated to Afan Oromo (local language) then again back to English to check its consistency. Two translators translated independently then they were brought together to manage any inconsistencies. Before data collection commenced, a pre-test on 5 % (*n* = 36) of the sample was performed in two kebeles that were out of the study area. Two full days of training were given to data collectors and every step was followed and supervised; questioner completeness was checked at the field and during data entry. The collected data were entered and verified using Epi-data software version 3.1. As much as possible efforts were made to enhance the recall ability of the mother and face-to-face interview was made in a separate, private area to avoid social desirability and recall bias.

### Data analysis

The data were checked for completeness and consistency before being entered into a computer. Then it was coded, entered and verified using EPI-DATA 3.1 software. Then entered data were exported to SPSS version 21 for analysis. Descriptive summaries like frequency, proportion, cross tabs and graphical presentations were made to describe the data. An independent sample *t*-test was performed to compare significant differences of variables among urban and rural children. Meanwhile, the normality of data distribution was checked.

Initially, the analysis of data was done by using bivariate linear regression to determine the association between dependent variables and predictors. Then significant variables (*P*-value ≤ 0⋅2) obtained by the bivariate analysis were included in the multivariate linear analysis. Assumptions of the linear regression model (normality, linearity, absence of multi-collinearity and homoscedasticity of error terms) were checked as described elsewhere^(27)^ and they were found to be satisfied. Model fitness was assessed using an adjusted *R*^2^ value. Final models were derived using the backward elimination method after excluding variables with a high variance inflation factor at a *P*-value of <0⋅05 level of significance Outputs of the analysis were provided in unstandardised regression coefficients (*β*).

## Results

### Socio-demographic characteristics of the respondents

A total of 674 pairs of child and mothers/caregivers were included in the study giving a response rate of 98⋅25 %. Among a total of respondents, 340 (50⋅4 %) were urban residents, whereas 334 (49⋅6 %) were rural residents. The mean (±sd) age of mothers/caregivers was 27⋅86 (±5⋅2) years with no significant difference in urban and rural residents, whereas the mean (±sd) age of children was 12⋅09 (±4⋅18) months in urban and 13⋅23 (±4⋅73) months in rural with a significant difference (*P* = 0⋅001). Around three-quarters of the children were from orthodox mothers/caregivers (from which nearly 40 % were urban), nearly 83 % were from married (of which 42 % were rural residents) and 70⋅2 % were from a housewife mother/caregiver. The median monthly income of the family was 2000 (IQR = 4000) Ethiopian Birr, and the mean (±sd) family size was 3⋅6 (±1⋅13) in urban and 4⋅27 (±1⋅62) in rural residents with a significant difference in family size (*P* < 0⋅001; Table 1).

**Table 1.** Socio-demographic characteristics of 6–23-month children and their mother/caregiver in the West Shewa zone, Oromia, Ethiopia, 2019

Variable	Categories	Residence				Total *N* 674	
		Urban, *n* 340		Rural, *n* 334			
		*n*	%	*n*	%	*N*	%
Age of mother/caregiver in years	15–24	91	13⋅5	104	15⋅4	195	28⋅9
	25–34	205	30⋅4	174	25⋅8	379	56⋅2
	35–44	44	6⋅5	56	8⋅3	100	14⋅8
Religion of mother/caregiver	Orthodox	269	39⋅9	219	32⋅5	488	72⋅4
	Protestant	33	4⋅9	89	13⋅2	122	18⋅1
	Muslim	38	5⋅6	17	2⋅5	55	8⋅2
	Others	–		9	1⋅3	9	1⋅3
Current marital status of mother/caregiver	Single	25	3⋅7	8	1⋅2	33	4⋅9
	Married	277	41⋅1	284	42⋅1	561	83⋅2
	Divorced	38	5⋅6	35	5⋅2	73	10⋅8
	Widowed	–		7	1⋅0	7	1⋅0
Educational status of mother or caregiver	No formal education	158	46⋅47	168	52⋅30	326	48⋅37
	Primary (1–8)	60	17⋅63	94	28⋅14	154	22⋅84
	Secondary (9–12)	49	14⋅42	46	13⋅77	95	14⋅10
	Attended college/university	73	21⋅47	26	7⋅8	99	14⋅7
Educational status of father or caregiver	No formal education	153	45⋅0	114	34⋅13	267	39⋅61
	Primary (1–8)	23	6⋅76	107	32⋅04	130	19⋅29
	Secondary (9–12)	69	20⋅30	63	18⋅9	132	19⋅6
	Attended college/university	95	27⋅9	50	15⋅0	145	21⋅5
Occupation of mother/caregiver	House wife	222	65⋅3	251	75⋅1	473	70⋅2
	Government employee	47	13⋅8	17	5⋅1	64	9⋅5
	Merchant	50	14⋅7	43	12⋅9	93	13⋅8
	Private employee	21	6⋅2	23	6⋅9	44	6⋅5
Occupation of father/caregiver	Farmer	125	36⋅5	195	58⋅4	320	47⋅5
	Government employee	67	19⋅7	46	13⋅8	113	16⋅8
	Merchant	35	10⋅3	38	11⋅4	73	10⋅8
	Private employee	113	33⋅2	55	16⋅5	168	24⋅9
Own farmland/garden	Yes	119	35⋅0	150	44⋅9	269	39⋅9
	No	221	65⋅0	184	55⋅1	405	60⋅1
Owned livestock	Yes	109	32⋅1	189	56⋅6	298	44⋅2
	No	231	67⋅9	145	43⋅4	376	55⋅8
Decision maker in the house	Mother only	63	18⋅5	78	23⋅4	141	20⋅9
	Father only	137	40⋅3	86	25⋅7	223	33⋅1
	Both mother and father	121	35⋅6	164	49⋅1	285	42⋅3
	Caregiver only	19	5⋅6	6	1⋅8	25	3⋅7
Family size	<5	292	85⋅9	225	67⋅4	517	76⋅7
	≥5	48	14⋅1	109	32⋅6	157	23⋅3

### Maternal and child health characteristics

The finding indicated that nearly 53 and 47 % of male and female children were urban and rural residents, respectively, with no difference in sex distribution among urban and rural settings. Around 60 % of the urban and 40 % of the rural children were first-order children with a significant difference (*P* < 0⋅001), a majority of the children found in the age group of 6–11 months with a significant difference in the age distribution of the children among urban and rural residents (*P* < 0⋅001). Nearly 19 % of the urban child and 21 % of the rural child were living with their mother only, whereas nearly 77 % of urban and rural children were living with their mother and father with no difference in living arrangements. Concerning ANC visits and bits of advice given for IYCF practices of the current child, more than 90 % of the mothers attended ANC with no difference in urban and rural, but only 50 % of urban and 56 % of rural residents received advice during their ANC visit making no significant difference among the settings. Moreover, around 68 % of urban and 82 % of the rural mothers attended Postnatal care (PNC), of which 15 % of urban and 28 % of rural residents received IYCF advice during their PNC visit with a significant difference in PNC service utilisation (*P* < 0⋅001; Table 2).

**Table 2.** Maternal and child health characteristics of child aged 6–23 months old and their counter mother/caregiver in the West Shewa zone, Oromia, Ethiopia, 2019

Variable	Categories	Residence				Total *N* 674	
		Urban, *n* 340		Rural, *n* 334			
		*n*	%	*n*	%	*N*	%
Sex of the child	Male	181	53⋅2	179	53⋅6	360	53⋅4
	Female	159	46⋅8	155	46⋅4	314	46⋅6
Age category of the child in months	6–11	210	61⋅8	155	46⋅4	365	54⋅2
	12–17	96	28⋅2	112	33⋅5	208	30⋅9
	18–23	34	10⋅0	67	20⋅1	101	15⋅0
Birth order of the child	First	206	60⋅6	134	40⋅1	340	50⋅4
	Second to fifth	132	38⋅8	183	54⋅8	315	46⋅7
	Sixth and above	2	0⋅6	17	5⋅1	19	2⋅8
Child lives with	Mother only	64	18⋅8	69	20⋅7	133	19⋅7
	Both mother and father	263	77⋅4	257	76⋅9	520	77⋅2
	Caregiver	13	3⋅8	8	2⋅4	21	3⋅1
Ever had ANC visit for current child	Yes	312	91⋅8	307	91⋅9	619	91⋅8
	No	28	8⋅2	27	8⋅1	55	8⋅2
IYCF practice advice during ANC visit	Yes	175	51⋅5	188	56⋅3	363	53⋅9
	No	165	48⋅5	146	43⋅7	311	46⋅1
Ever had PNC visit for current child	Yes	232	68⋅2	276	82⋅6	508	75⋅4
	No	108	31⋅8	58	17⋅4	166	24⋅6
IYCF practice advice during PNC visit	Yes	51	15⋅0	95	28⋅4	146	21⋅7
	No	289	85⋅0	239	71⋅6	528	78⋅3

### Infant/young child feeding practices

Almost all of the urban and rural residents have been breastfeeding their children, of which 70⋅0 % of the urban and rural residents had started breastfeeding immediately upon the birth of the child but 8⋅2 and 15⋅3 % of urban and rural residents had started breastfeeding after an hour, respectively, with a significant difference in the initiation of EBF among urban and rural settings (*P* < 0⋅001). The mean (±sd) age at initiation of complementary feeding was 5⋅36 (±1⋅48) and 5⋅70 (±0⋅93) months in urban and rural settings with a significant difference in the initiation of complementary feeding (*P* < 0⋅001; Table 3).

**Table 3.** Infant and young child feeding characteristics of child aged 6–23 months old and their counter mother/caregiver in the West Shewa zone, Oromia, Ethiopia, 2019

Variable	Categories	Residence				Total *N* 674	
		Urban *n* 340		Rural *n* 334			
		*n*	%	*n*	%	*N*	%
Ever breastfed the child	Yes	340	50⋅4	334	49⋅6	674	100
	No	–		–		–	
Early initiation of Exclusive Breastfeeding	Immediately	238	70⋅0	233	69⋅8	471	69⋅9
	After 1 h	28	8⋅2	51	15⋅3	79	11⋅7
	After 2 h	10	2⋅9	14	4⋅2	24	3⋅6
	After more than 2 h	64	18⋅8	36	10⋅8	100	14⋅8
Child started complementary feeding	Yes	340	50⋅4	334	49⋅6	674	100
	No	–		–		–	
Gave food 1 d prior to the study at day/night	Yes	340	50⋅4	334	49⋅6	674	100
	No						
Daily frequency of food given	Twice	18	5⋅3	57	17⋅1	75	11⋅1
	Three times	106	31⋅2	112	33⋅5	218	32⋅3
	Four or more times	216	63⋅5	165	49⋅6	381	56⋅5
Source of food at the household level	Own production, hunting, gathering, fishing	84	24⋅7	186	55⋅7	270	40⋅1
	Purchased	340	50⋅4	250	74⋅9	590	87⋅5
	Borrowed, bartered, exchanged for labour, gifted	–		5	1⋅5	5	0⋅7
Means of transportation to the market	Vehicle (Taxi, Bajaj)	226	66⋅5	158	47⋅3	384	57⋅0
	Horse, cart	–		24	7⋅2	24	3⋅6
	On foot	213	62⋅6	225	67⋅4	438	65⋅0
Minimum DDS	Achieved	60	17⋅6	116	34⋅7	176	26⋅1
	Not achieved	280	82⋅4	218	65⋅3	498	73⋅9
Minimum meal frequency	≥4 times/d	216	63⋅5	165	49⋅4	381	56⋅5
	<4 times/d	124	36⋅5	169	50⋅6	293	43⋅5

**Fig. 1.**
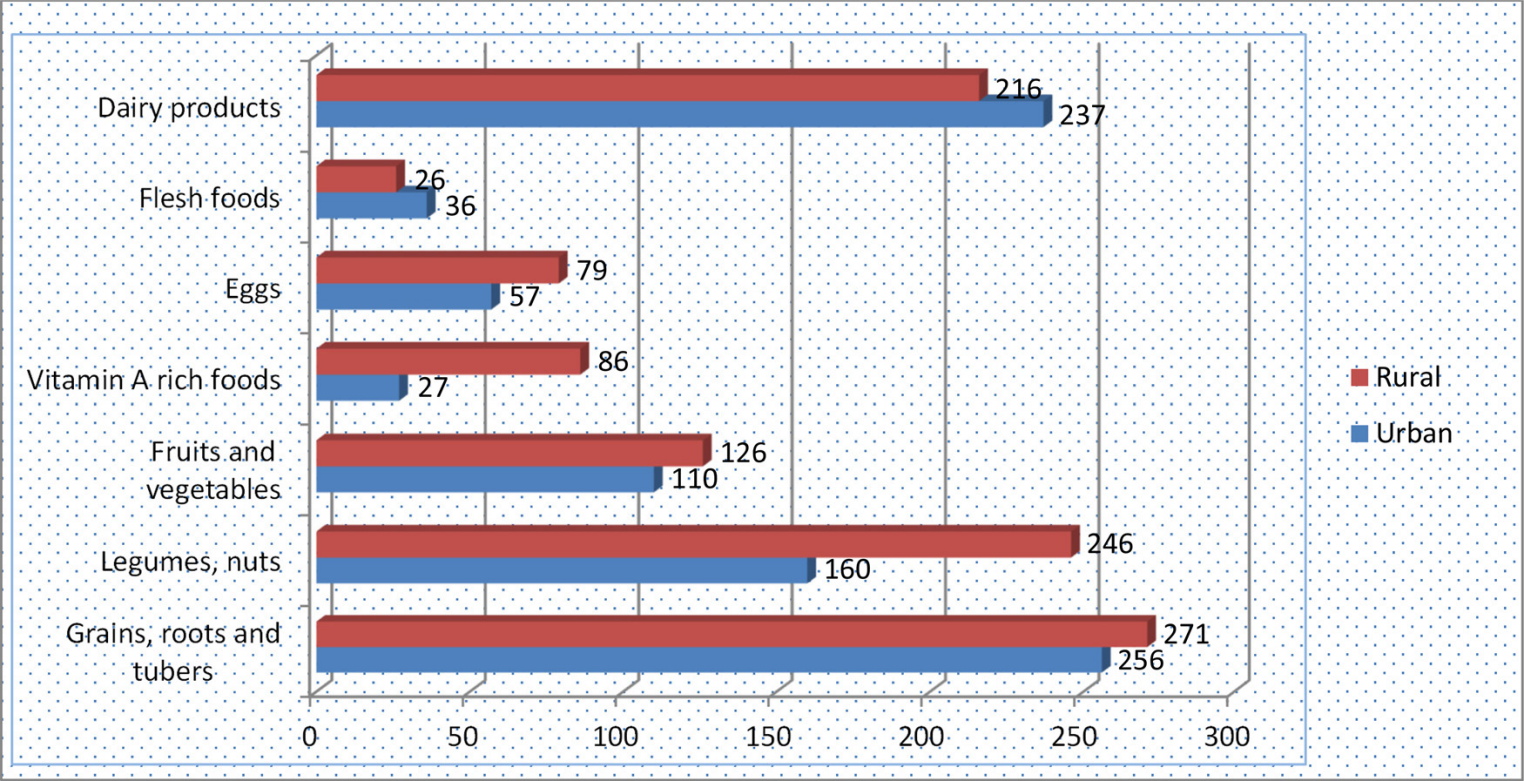
Food groups consumed by children aged 6–23 months old in urban and rural settings, West Shewa, Oromia, Ethiopia, 2019.

Seven food groups were assessed among urban and rural settings to calculate some of the core indicators for IYCF practices. The finding indicated that grains, roots and tuber groups were the most commonly fed food groups for children in both urban (38⋅0 %) and rural (40⋅2 %) settings with no difference (*P* = 0⋅081); similarly, there was no difference in the utilisation of milk and milk products in urban (35⋅2) and rural (32⋅0) (*P* = 0⋅190). The finding identified that there was a difference in the utilisation of pulse [urban (23⋅7 %), rural (36⋅5), *P* < 0⋅001], vitamin A-rich food items [urban (4⋅0 %), rural (12⋅8), *P* < 0⋅001] and eggs [urban (8⋅5 %), rural (11⋅7 %), *P* = 0⋅003] as described in Fig. 1.

Based on the MDD score (a minimum of four food items from seven food groups), 8⋅9 % of the urban and 17⋅2 % of the rural children achieved their minimum DDS with an overall dietary diversity of 26⋅1 % (95 % CI 22⋅8, 29⋅5). The difference in both settings was significant at *P* < 0⋅001. Furthermore, 32⋅0 % of urban and 24⋅5 % of rural residents provided their children with four or more meal frequencies (MMF) per day, and the overall MMF was 56⋅5 % (95 % CI, 52⋅7, 60⋅2) with a statistically significant difference (*P* < 0⋅038) in both settings.

### Factors associated with a level of dietary diversity

Factors like a child from a merchant mother, own production of foods at a household level, frequent advice of IYCF practices during PNC visit and continued breastfeeding after 6 months of age were positively associated with an increased level of dietary diversity at urban residents. Factors like increased child's age in months, frequent meal per day, maternal secondary educational level, living with caregiver only, having a merchant father, advice of IYCF practice during PNC visit and the utilisation of horse as a means of transportation were positively associated with the level of dietary diversity at rural residents (Table 4).

**Table 4.** Multivariable linear regression results of 6–23-month-old children and mother/caregiver residing in urban and rural parts of the West Shewa zone, Oromia, Ethiopia, 2019

Variable	Categories	Residence					
		Urban, *N* 340			Rural, *N* 334		
		*β*	95% CI	*P*-value	*β*	95% CI	*P*-value
Intercept		1⋅08	0⋅68, 1⋅48	<0⋅001	2⋅28	1⋅58, 2⋅97	<0⋅001
Educational status of mother	No formal education ^Ref^						
	Primary education	−0⋅34	−0⋅64, −0⋅03	0⋅033^a^			
	Secondary education				0⋅80	0⋅41, 1⋅19	<0⋅001^a^
Educational status ofFather	No formal education ^Ref^						
	Secondary education	0⋅27	−0⋅02, 0⋅57	0⋅071			
Marital status	Single	−0⋅59	−1⋅12, −0⋅06	0⋅029^a^			
	Married ^Ref^						
Child lives with	Both father and mother ^Ref^						
	Mother only	0⋅36	−0⋅01, 0⋅74	0⋅059			
	Care giver only				1⋅50	0⋅62, 2⋅39	0⋅001^a^
Frequency of meals per day					0⋅19	0⋅08, 0⋅31	0⋅001^a^
Childs aged in month					0⋅06	0⋅03, 0⋅09	<0⋅001^a^
Duration of continuous breast feeding after 6 months		0⋅09	0⋅07, 0⋅12	<0⋅001^a^			
Occupation of mother	Housewife				−0⋅49	−0⋅81,−1⋅16	0⋅003^a^
	Merchant	0⋅58	0⋅31, 0⋅845	<0⋅001^a^			
	Government employee ^Ref^						
Occupation of father	Merchant				0⋅52	0⋅10, 0⋅94	0⋅016^a^
	Government employee ^Ref^						
Frequent number of ANC visits					−0⋅09	−0⋅16, −0⋅01	0⋅022^a^
Frequent number of PNC visits		−0⋅55	−0⋅84, −0⋅26	<0⋅001^a^			
Advice of IYCF practice during PNC visit		0⋅16	0⋅095, 0⋅23	<0⋅001^a^	0⋅40	0⋅09, 0⋅72	0⋅013^a^
Decision maker at home	Mother only	−0⋅77	−1⋅15, −0⋅39	<0⋅001^a^			
	Caregiver	−0⋅62	−1⋅24, 0⋅01	0⋅053			
	Both father and mother ^Ref^						
Source of daily food	Own production	0⋅59	0⋅27, 0⋅91	<0⋅001^a^			
	Purchased				−0⋅42	−0⋅74, 0⋅10	0⋅01^a^
	Others (borrowed, aided) ^Ref^						
Means of transportation to collect daily food	Horse				0⋅90	0⋅36, 1⋅45	0⋅001^a^
	Other means ^Ref^						

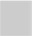
The shaded box indicates that variables under the respective row are not applicable for either to the urban or rural category.

Ref indicates a reference group.

*β* indicates the unstandardised coefficient for both models under urban and rural categories.

a This indicates a statistically significant variable with a *P*-value of <0⋅05.

## Discussion

The study revealed that the overall proportion of adequate DDS (based on consumption of four or more food groups on daily basis 24 h before the study) was 26⋅1 % (95 % CI 22⋅8, 29⋅5), from which 8⋅9 % were urban residents and 17⋅2 % were rural residents. This finding is consistent with prior findings that were conducted in different parts of the country (Ethiopia) ^(10,13,14,16)^. The finding of dietary diversity proportion is higher than studies conducted in Dabat (17 %), Gorche (10⋅6 %), Sinana district (13 %) and Jimma zone (16⋅1 %)^(12,18,21,22)^. The difference might be attributed to variations in socio-demographic, economic culture since two of the studies were from the northern part, whereas one was from the southwest and southern part of Ethiopia. Furthermore, the difference might be due to slight variations in sample size, design and study setting in which some of the studies were conducted in purely urban or rural but some enrolled a mix of urban and rural residents. Contrary to this, the finding in the present study was lower than a study conducted in Addis Ababa, the capital of Ethiopia (59⋅9 %)^(20)^. The difference might be attributed to study setting and sample size since the present study is community-based comparative with a higher sample size, whereas the former was institution-based. Further studies are also suggesting that infant and young children in the capital (Addis Ababa) or in urban had been receiving a more diversified daily meal leading to a higher DDS^(9,28)^.

The study also concluded that the overall proportion of children who achieved their MMFs (consumption of daily meals four or more times per day) was 56⋅5 % (95 % CI 53⋅0, 60⋅4), of which 32⋅0 % of them were urban and 24⋅5 % of them were rural residents. This finding was comparable to studies conducted in Addis Ababa (55⋅1 %) and Dangila (50⋅4 %)^(20,29)^. But the finding of the present study was lower than studies conducted in the Sidama zone and Wolaita Sodo (68⋅9 %)^(22,30)^.

The present study also tried to identify factors that affect the level (either increasing or decreasing) of dietary diversity among 6–23-month-old children residing in urban and rural settings using linear regression.

In previous studies, a number of factors had been identified as predictor variables of dietary diversity but failed to show to what extent each variable contributes either in increment or reduction of levels of dietary diversity. However, in the present study also a number of variables were identified with their extent of influence on the level of dietary diversity both for urban and rural residents.

The present study revealed that with one unit (grade) increase in secondary education of mother, there would be a 0⋅80 (95 % CI 0⋅41, 1⋅19) unit increment in the level of dietary diversity of child in a rural setting, whereas being in primary education of mother would reduce dietary diversity by 0⋅34 (95 % CI −0⋅64, −0⋅03) when compared with mothers with no formal education keeping other factors constant. This finding is supported by findings from other studies in which maternal education is the most predictor variable of dietary diversity^(13,16,20,29,31)^. Paternal education also contributes a lot to the utilisation of diversified daily meals. But this finding failed to show the effect of a father's education on the level of dietary diversity both in urban and rural settings.

Marital status can also contribute to the utilisation of diversified daily meals for children. The present study revealed that being single reduces dietary diversity by 0⋅59 (95 % CI −1⋅12, −0⋅06) when compared with married mothers in urban settings keeping other factors constant. But the study did not support the effect of marital status on the level of dietary diversity in rural settings. Living arrangements are nowadays contributing to a number of health events. On contrary, the present study witnessed that child's living arrangement did not significantly affect their dietary level. For children who are supposed to live with caregiver, their dietary diversity increases by 1⋅5 (95 % CI 0⋅62, 2⋅39) keeping other factors constant in rural residents. Children's living arrangement has no significant effect at all on urban residents. This might be due to the fact that in urban whether a mother or a father or both physically exist or not, a caregiver or any other substitute (e.g. attendants in kinder garter) can give care and feed the child what the family provided.

The age of the child also plays a role related to the level of dietary diversity. The present study indicated that a 1-month increase in the age of the child would result in a 0⋅06 (95 % CI 0⋅03, 0⋅09) unit increase in the level of dietary diversity in rural settings. This finding is comparable with some studies conducted in Ethiopia^(14,29)^. This positive relationship can be justified by the fact that as age increases, food preference and demand also increase due to physiological changes and growth. These would result in the increased frequency of daily meals that again facilitate the increased utilisation of a variety of meals. But an increased age has no relation with levels of dietary diversity in an urban setting.

Occupation of both mother and father has also a significant role in the provision of a diversified diet at home. The present study revealed that being a housewife would reduce levels of dietary diversity by 0⋅49 (95 % CI −0⋅81, −1⋅16) when compared with government employee mothers at rural settings, and also being a merchant mother could increase levels of dietary diversity of their children by 0⋅58 (95 % CI 0⋅31, 0⋅85) when compared with government employee at urban. This finding is comparable with findings from different parts of the country (Ethiopia)^(13,32)^. This is justified by the fact that mothers who had their own income through different jobs (like merchant) can easily access and gather a variety of food for their children but housewife (housemaid) mother's life is mostly dependent on their husband so they failed to collect daily meal as per their need. This could directly affect the dietary diversity of their children. Additionally, a merchant father also could increase levels of dietary diversity by 0⋅52 (95 % CI 0⋅10, 0⋅94) when compared with government-employed father at rural. The effect of father's occupation on dietary diversity was also witnessed in different studies^(14,30)^.

Antenatal and postnatal care visits to the health facility and receiving advice on IYCF practices were also showing significant association with the dependent variable. Mothers/caregivers who received at least one advice related to IYCF practice would increase their children's level of dietary diversity by 0⋅16 (95 % CI: 0⋅10, 0⋅233) in urban settings and 0⋅40 (95 % CI 0⋅09, 0⋅72) in rural settings. This finding is supported by studies conducted elsewhere^(10,18,21)^. This is due to the fact that advice related to IYCF practice, especially if supported by demonstration, would increase mothers/caregivers’ knowledge and practice on food selection, cooking and serving capacity so that their children would get sufficient and frequent diversified meals at daily basis^(15,16,31)^. Furthermore, decisions made by mother only reduce levels of dietary diversity by 0⋅77 (95 % CI −1⋅15, −0⋅39) when compared with decisions made by both mother and father in urban settings, whereas decisions made by caregiver only reduce levels of dietary diversity by 0⋅62 (95 % CI −1⋅24, 0⋅01) when compared with decisions made by both mother and father in urban residents. This finding is consistent with other findings^(10,21)^. But the study failed to show any association in rural settings.

The source of daily food can also contribute a huge role to levels of dietary diversity. It is logical that urban residents are mostly dependent on purchased or aided foods, whereas rural residents are mostly dependent on their own produced agricultural products. However, own production of daily consumed food (e.g. fruits and vegetables) would increase the variety of daily dishes in urban. This finding supports the above scenario in that own production of daily food would increase levels of dietary diversity by 0⋅59 (95 % CI 0⋅27, 0⋅91) when compared with other sources of food (borrowed, aided) in urban settings, whereas more purchased foods are utilised at rural settings, a −0⋅42 (95 % CI −0⋅74, −0⋅10) unit reduction in levels of dietary diversity would be the result. The finding is consistent with studies elsewhere^(21,29)^. Finally, the present study indicated that the utilisation of horse to collect or buy daily food increases levels of dietary diversity by 0⋅9 (95 % CI 0⋅36, 1⋅45) in rural settings when compared with other means of transportation.

### Strength and limitation of the study

Generally, the present study identified a number of factors that would enhance or reduce levels of dietary diversity with their direct individual magnitude of effect on a dependent variable. The study used a linear regression model to show the relationship between predictor and dependent variables. But, the present study failed to show the relationship of maternal knowledge on IYCF practice, cooking demonstration and household food security level with the dependent variable. Additionally, though training was given to data collectors and supervisors, and the objective of the present study was well communicated to mothers and caregivers of the children, recall bias and social desirability bias were among the limitation of the study.

## Conclusions and recommendations

In general, infant and young children who received the recommended dietary diversity and the MMF were low in the study area both in urban and rural settings. Furthermore, a significant difference was observed among children aged 6–23 months old who reside in urban and rural parts of the study area in terms of consumption of diversified diet and MMF. A number of factors were identified that contribute to a reduced level of dietary diversity practice among children aged 6–23 months old in urban and rural settings of the West Shewa zone, Oromia, Ethiopia.

Qualified antenatal and postnatal care services provided with adequate, demonstrated advice and counselling of mothers regarding IYCF practice are expected from health professionals and HEWs. Educational sectors shall better facilitate adult learning strategies for both mothers and fathers since an increased educational level plays paramount significance in changing the life of an individual and their offspring. Decision-making by both mother and father should be practiced at the community level both in urban and rural settings. Urban production of garden vegetables and fruits shall be practiced.
